# Supplementary figures and images for: High-resolution comparative atomic structures of two Giardiavirus prototypes infecting G. duodenalis parasite
Source: PLoS Pathog. 2024 Apr 10;20(4):e1012140. doi: 10.1371/journal.ppat.1012140 (PMC11081498; doi:10.1371/journal.ppat.1012140)

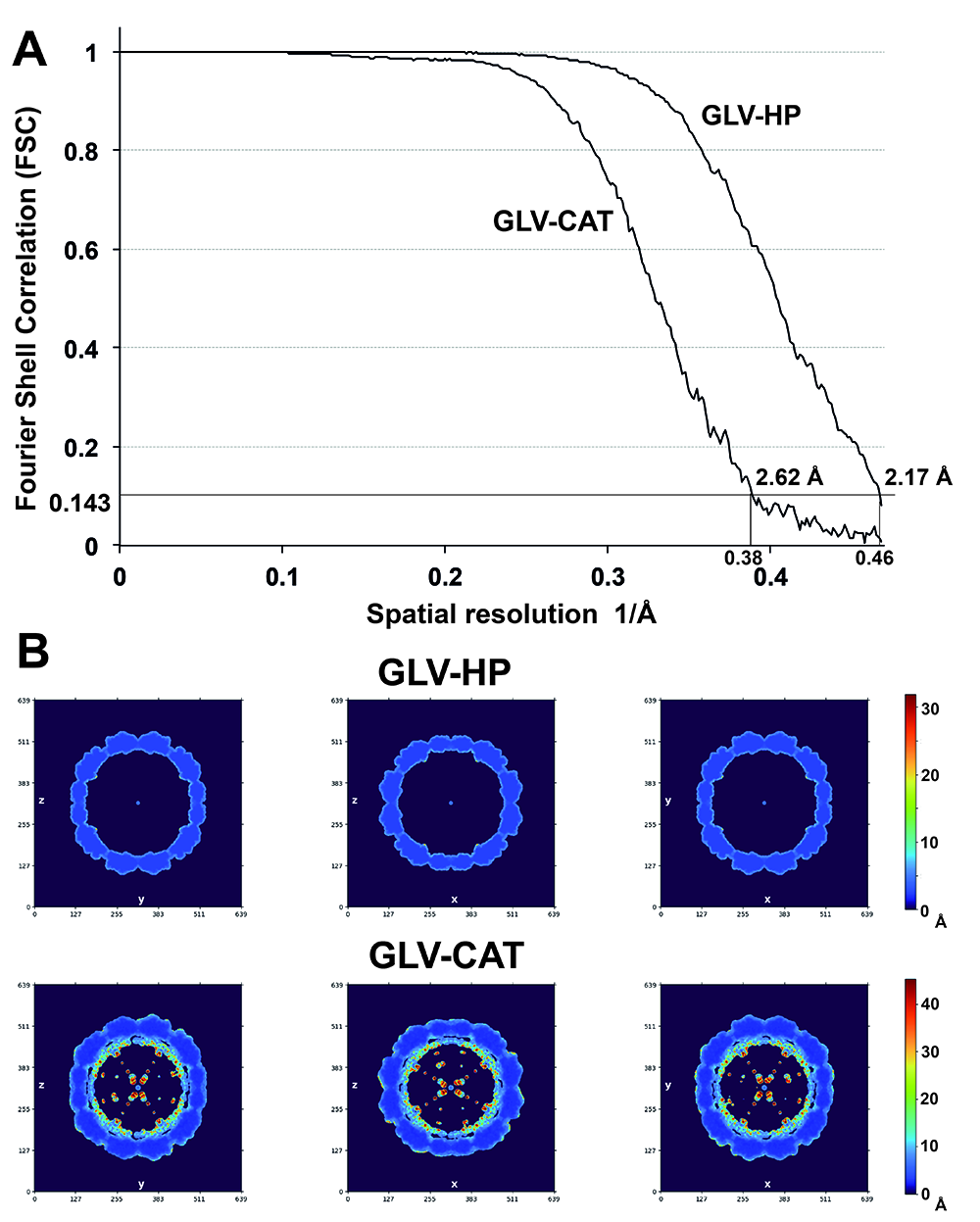

Supplement: S1 Fig — (A) FSC curves and (B) local resolution of the final cryo-EM 3D reconstruction for GLV-HP and GLV-CAT. (TIF) [file ppat.1012140.s001.tif]

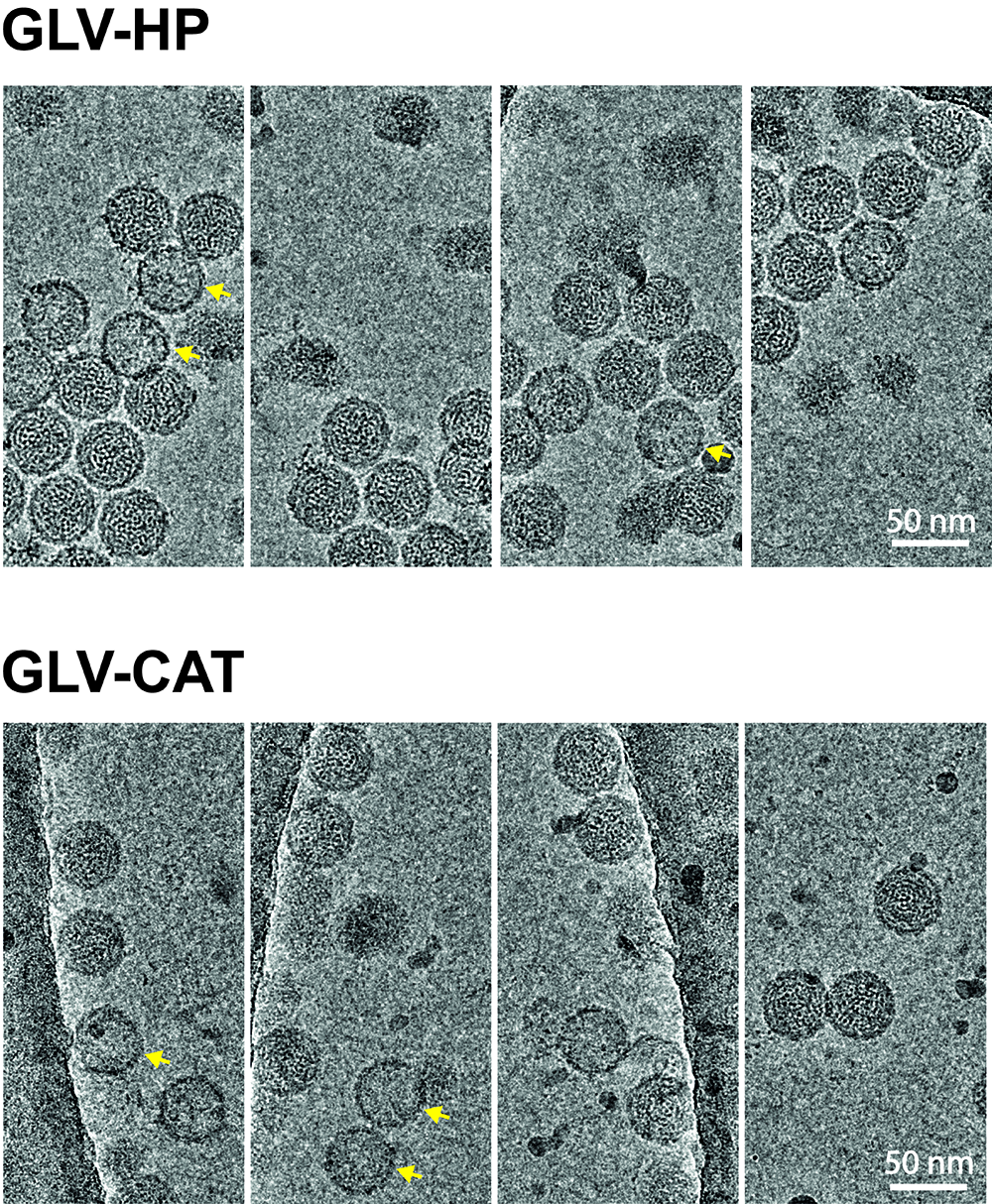

Supplement: S2 Fig — Yellow arrows indicate empty particles. (TIF) [file ppat.1012140.s002.tif]

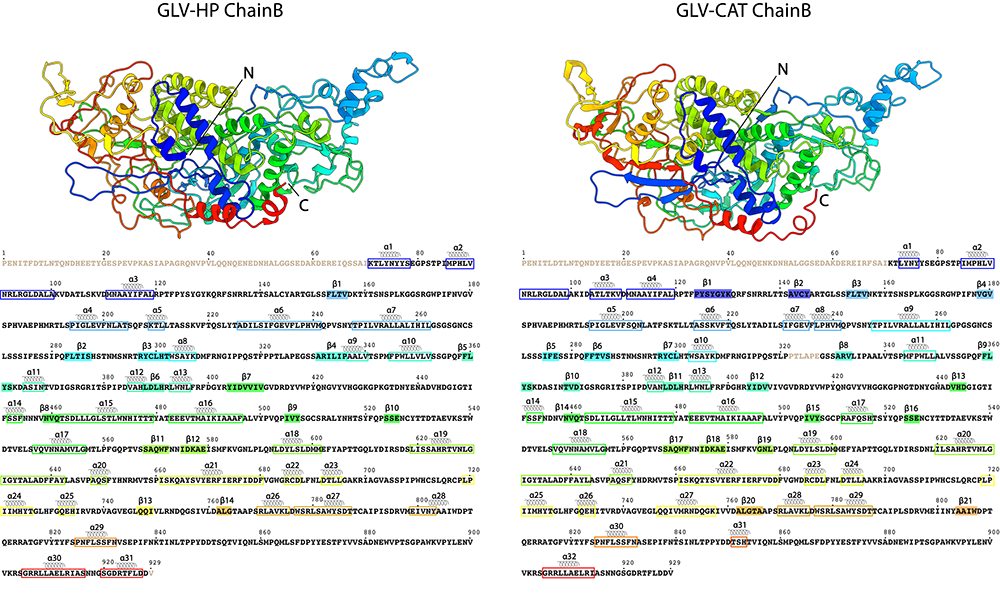

Supplement: S3 Fig — The rainbow color begins with blue at the N-terminus (Pro1) to red at the C-terminus (Val929). Considering the predicted internal IRES sequence in the GLV genome, the translation of ORF1 (CP) does not initiate from the first methionine but from an internal amino acid residue. The first amino acid residue of the CP was started from the internal Pro residue (PENIT …), according to a previous mass spectrometry analysis of the purified GLV particles. (TIF) [file ppat.1012140.s003.tif]

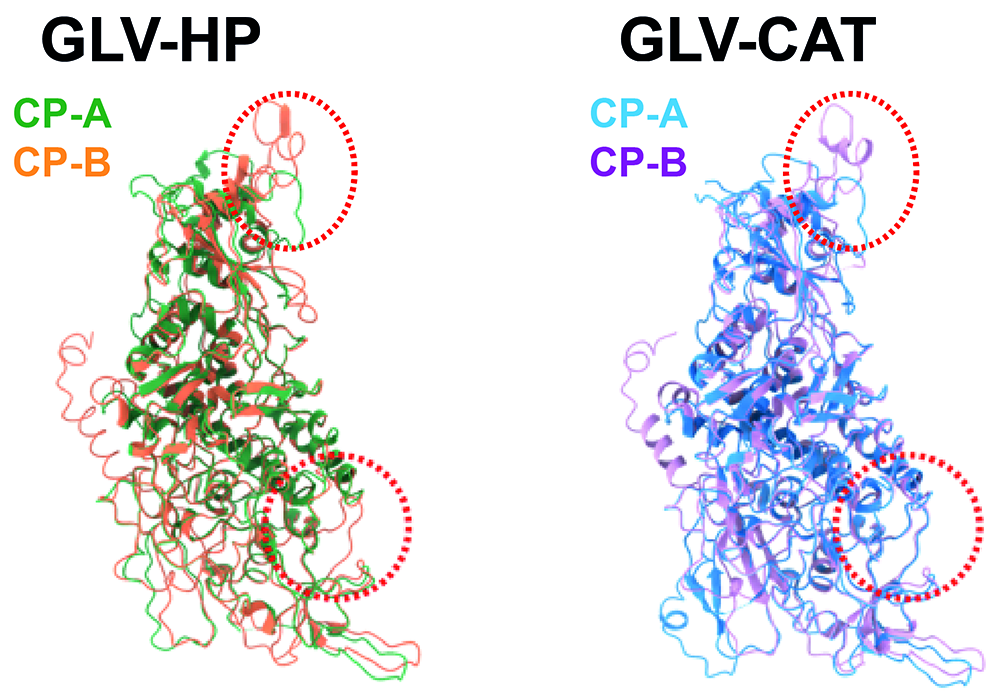

Supplement: S4 Fig — The CP-A and CP-B of GLV-HP are colored in green and orange, and those of GLV-CAT are colored in light blue and purple. The total RMSD values between CP-A and CP-B were calculated for GLV-HP and GLV-CAT. Some conformational changes are observed in the regions indicated by red dotted circles. The major core domain apart from N- and C-termini, and red dotted circled regions, are well aligned with RMSD = 0.618 Å over 684 Cα pairs between GLV-HP CP-A and CP-B), and 0.618 Å over 732 Cα pairs between GLV-CAT CP-A and CP-B while other loops and interfaces contribute to the overall higher RMSD values (4.338 Å across all 775 Cα pairs in GLV-HP, and 8.863 Å across all 848 Cα pairs in GLV-CAT). (TIF) [file ppat.1012140.s004.tif]

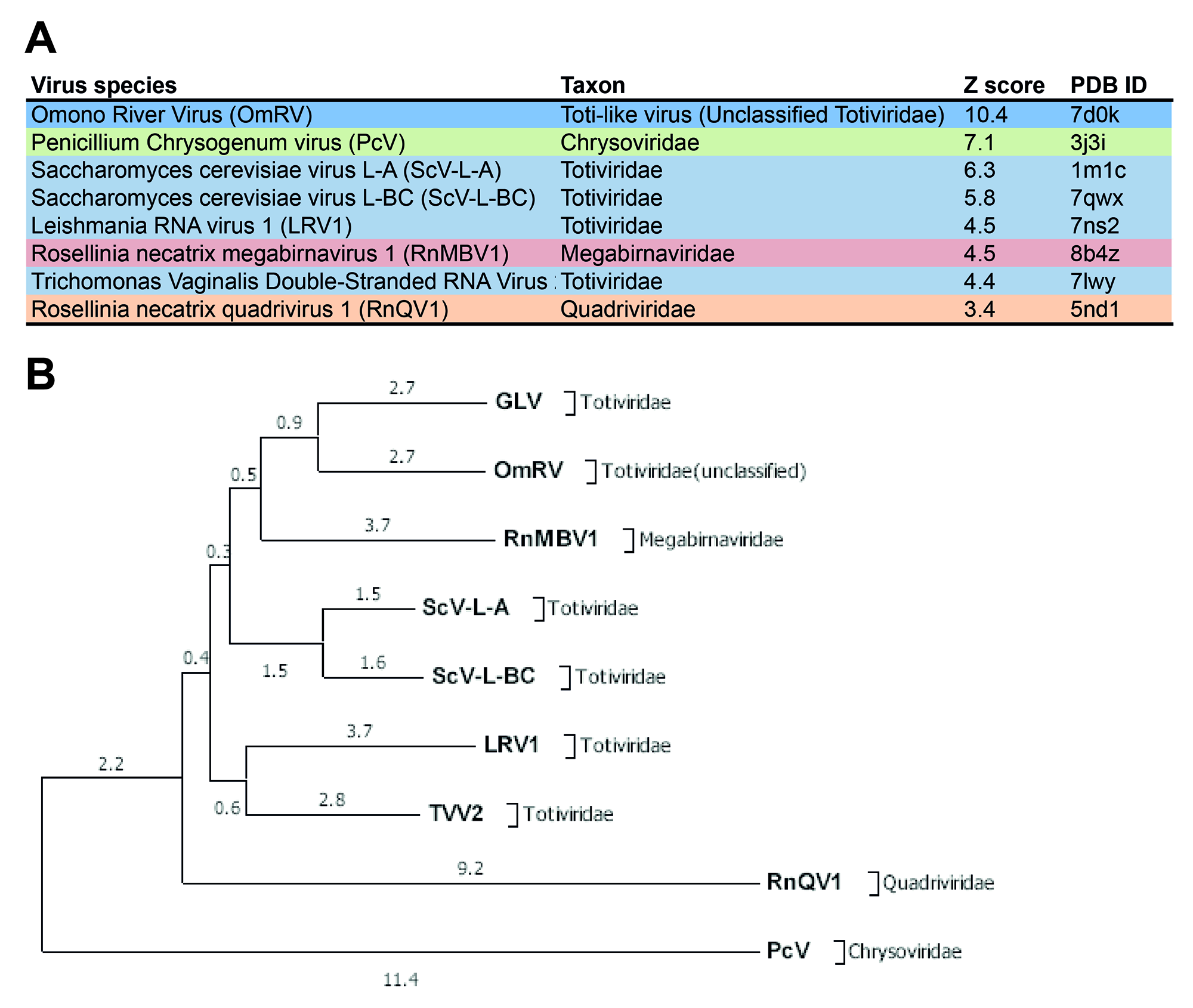

Supplement: S5 Fig — (A) List of the eight identified CPs that are similar to the GLV CP obtained from a Dali search (Z score: 3.4–10.4). (B) RMSD-based structural phylogeny generated by the alignment of the GLV CP with the eight CP structures in a Dali search. (TIF) [file ppat.1012140.s005.tif]

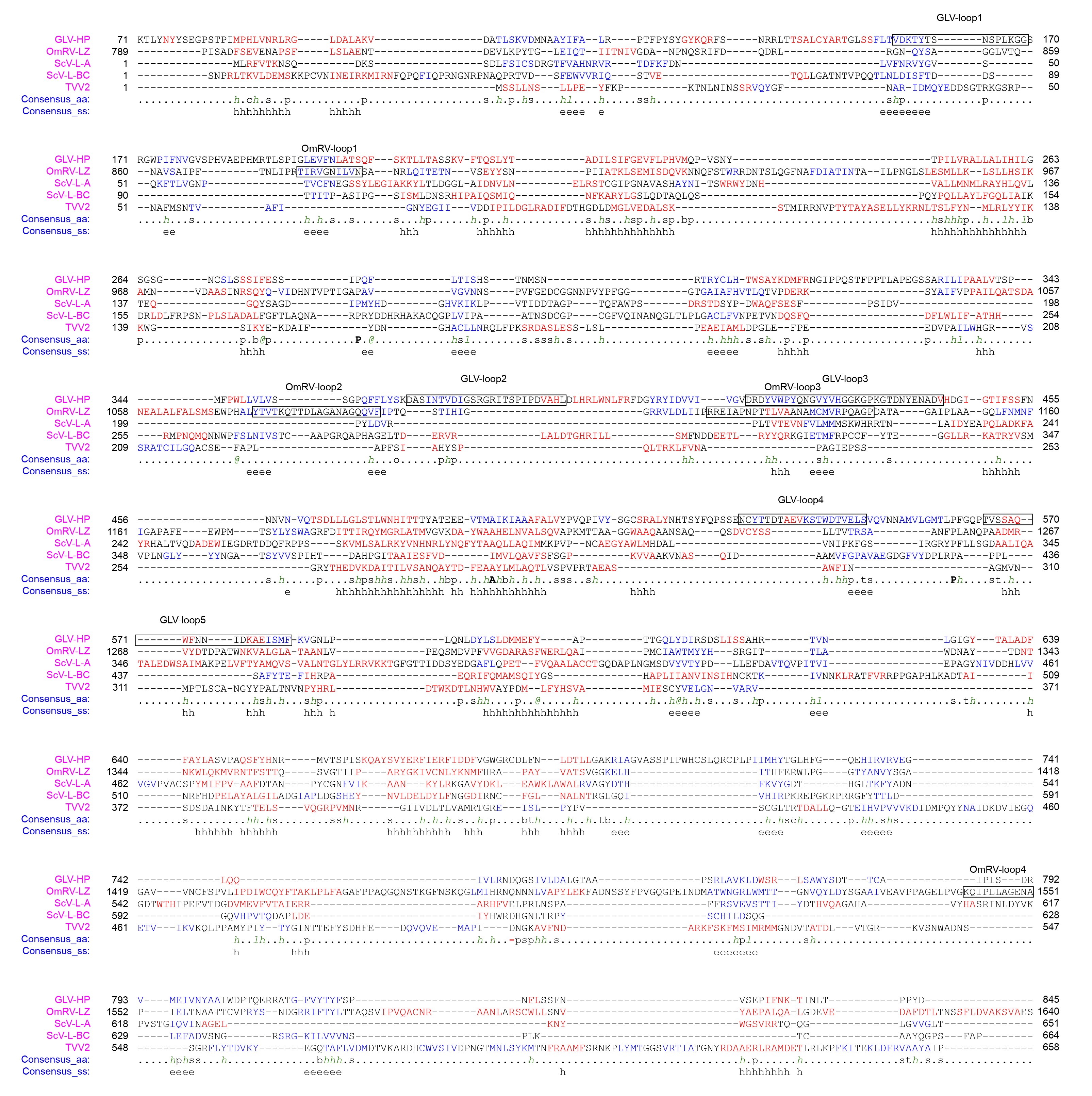

Supplement: S6 Fig — Amino acid sequences of GLV-HP (PDB ID: 8R0F, chain A), OmRV-LZ (PDB ID: 7D0K, chain A), ScV-L-A (PDB ID: 1M1C, chain A), ScV-L-BC (PDB ID: 7QWX, chain A), and TVV2 (PDB ID: 7LWY, chain A) CPs were aligned using PROMALS3D multiple sequence and structure alignment server. Extra surface loops of CP in GLV (loops 1–5) and OmRV (loops 1–4) are boxed in the aligned sequences. (TIF) [file ppat.1012140.s006.tif]

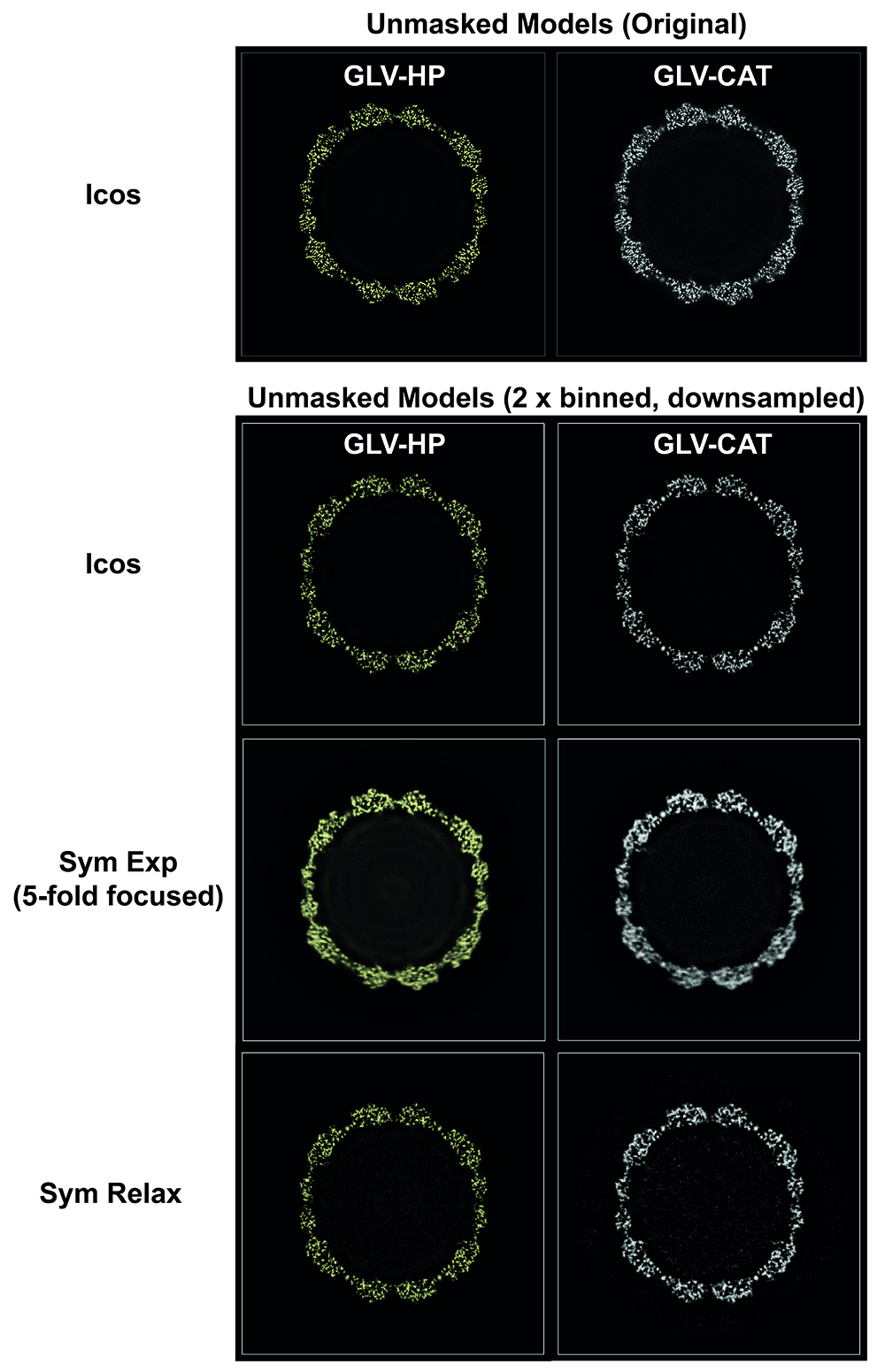

Supplement: S7 Fig — All images were generated from unmasked models. Asymmetric reconstructions were generated using symmetry expansion and local reconstruction options (Sym Exp) or the symmetry relaxation option (Sym Relax) in cryoSPARC software. A mask that covered one 5-fold vertex of the virus was utilized for the local reconstruction. The box size of the central slice is 640 x 640 pixels (1.06 Å/pixel) in the reconstruction with original particle images or 320 x 320 pixels (2.12 Å/pixel) in the reconstruction with downsampled particle images. (TIF) [file ppat.1012140.s007.tif]

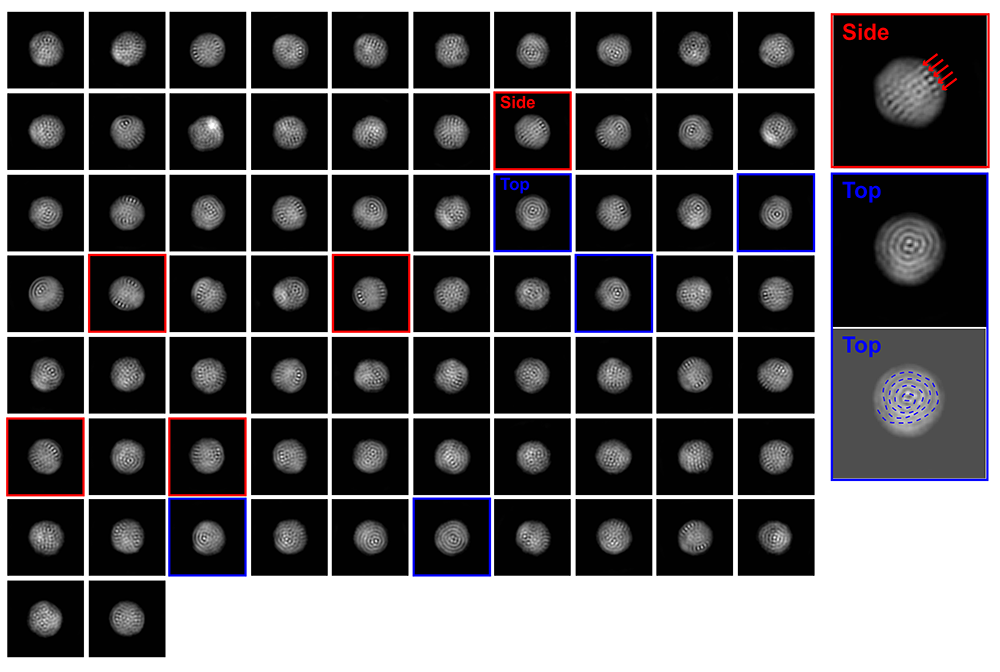

Supplement: S8 Fig — The striped genome (side views, red squares as examples) and the spiral genome (top views, blue squares as examples) are observed, which are typical 2D classes of a partially spooled genome organization in Reoviridae viruses. (TIF) [file ppat.1012140.s008.tif]

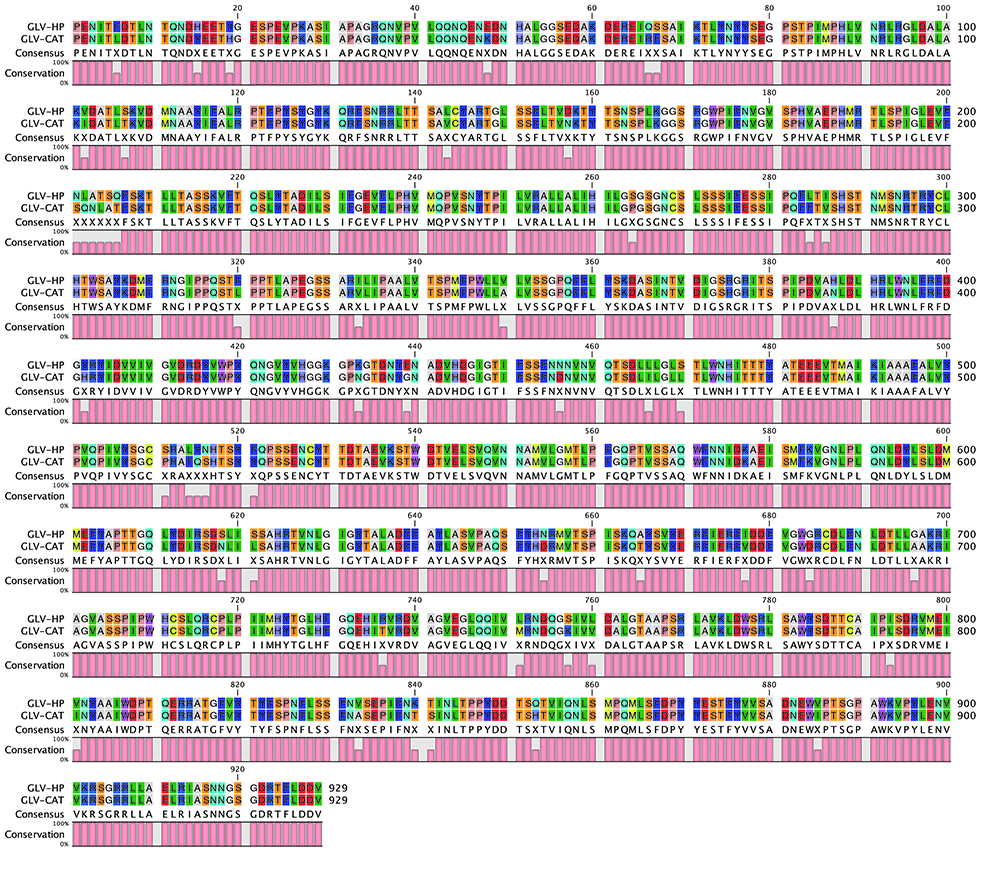

Supplement: S9 Fig — The alignment was generated by CLC Sequence Viewer 7.0. (TIF) [file ppat.1012140.s009.tif]

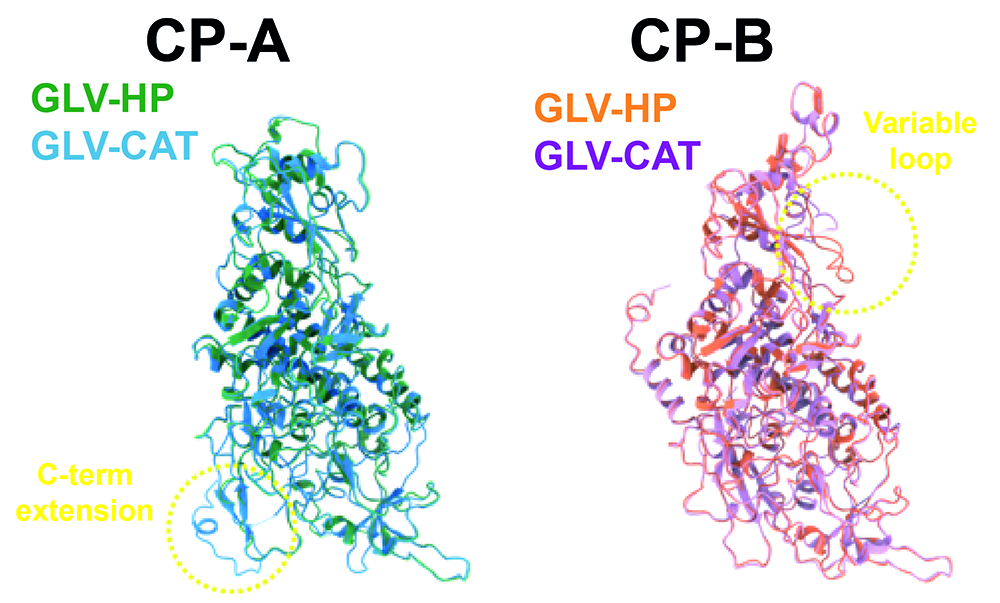

Supplement: S10 Fig — The overall RMSD values were calculated to evaluate the structural similarity. The major core domain are well aligned with RMSD = 0.401 Å across 773 Cα pairs between CP-As of GLV-HP and GLV-CAT, and 0.396 Å across 835 Cα pairs between CP-Bs of GLV-HP and GLV-CAT. Conformational changes were observed in the regions of C-terminal extension in CP-A and variable loop in CP-B, as indicated by yellow dotted circles. (TIF) [file ppat.1012140.s010.tif]

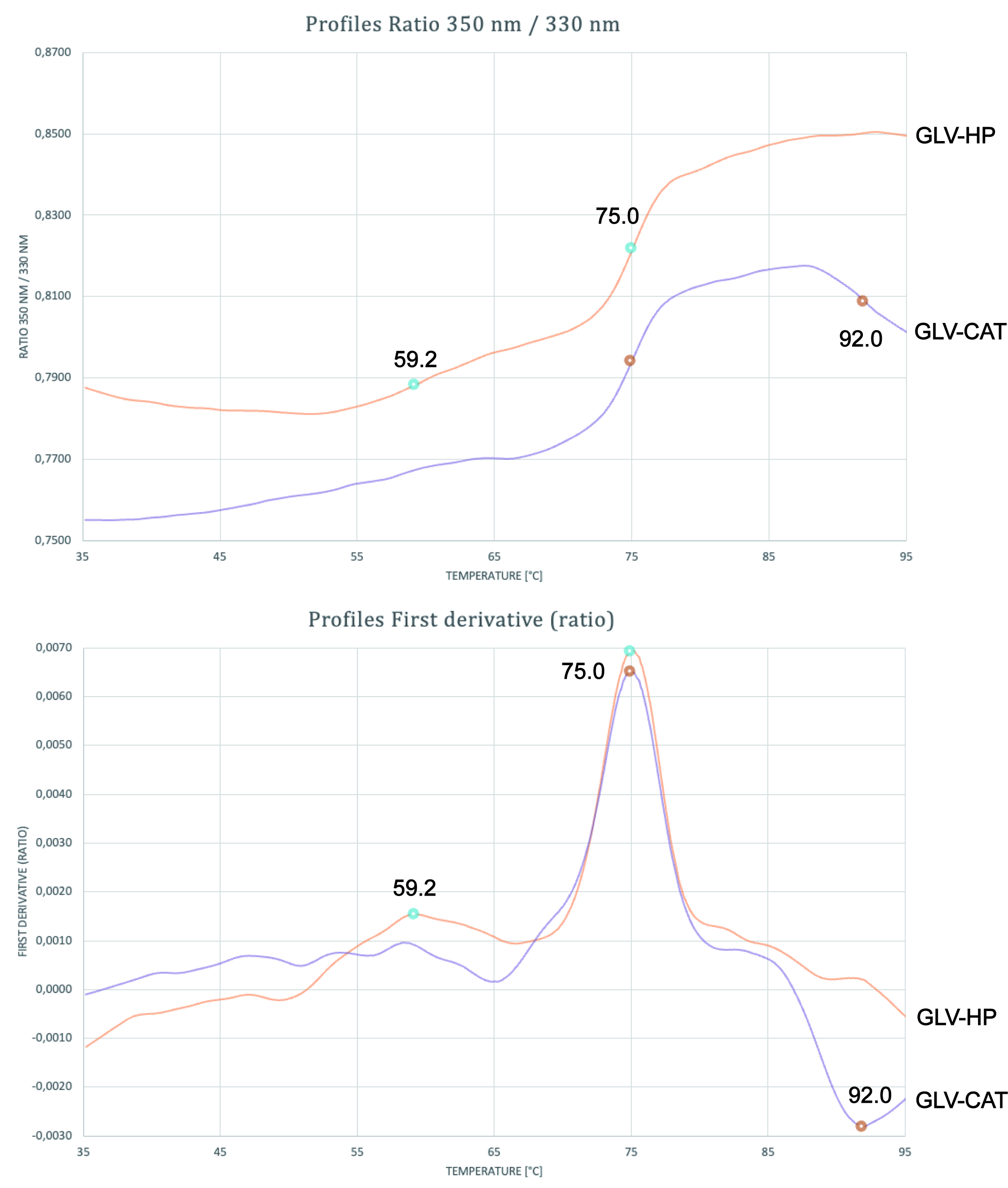

Supplement: S11 Fig — The thermographs and Ti values were obtained from purified samples of GLV-HP and GLV-CAT using Tycho NT.6 (NanoTemper). (TIF) [file ppat.1012140.s011.tif]

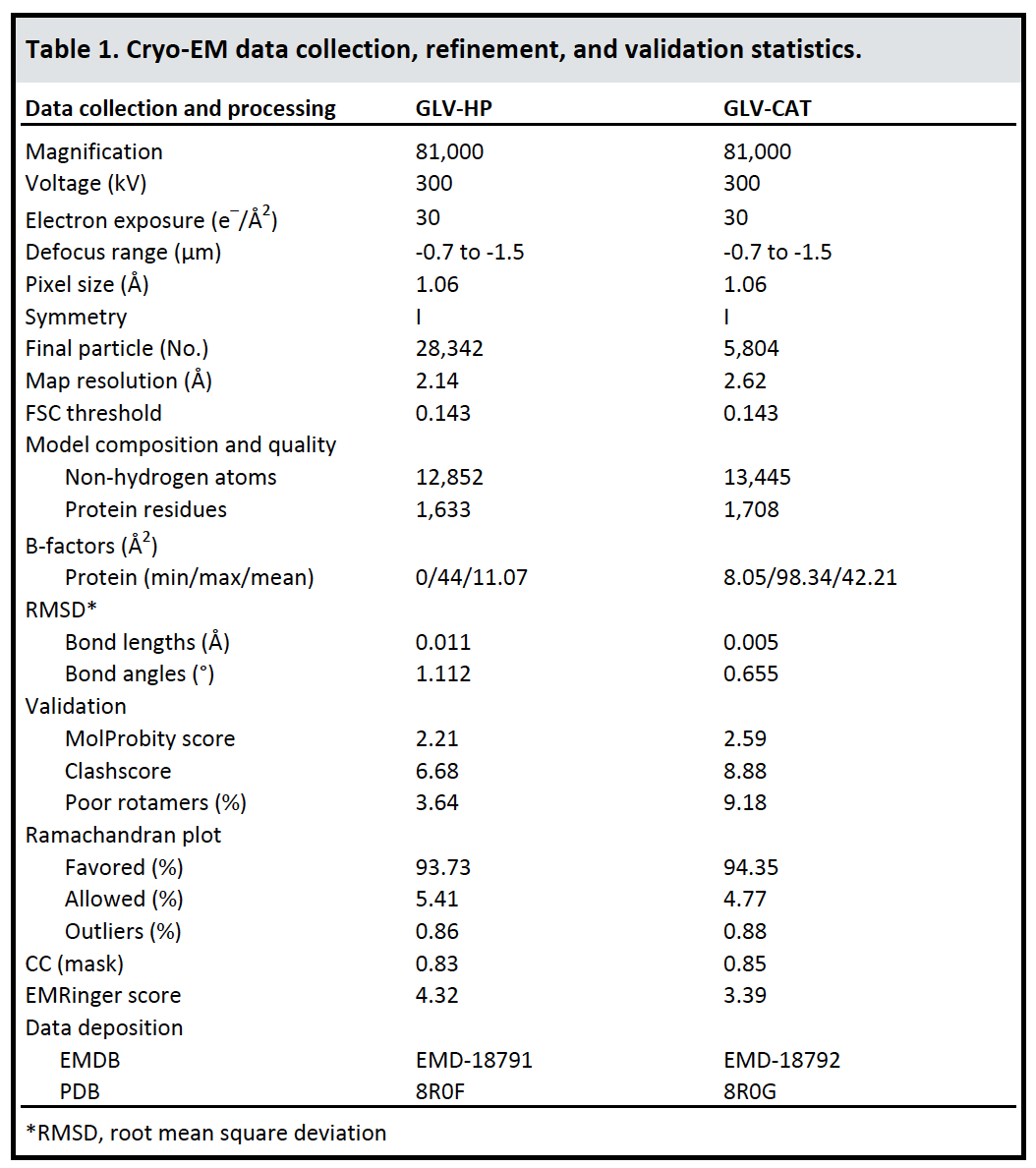

Supplement: S1 Table — (TIFF) [file ppat.1012140.s012.tiff]
