# Supplementary material for: High-resolution comparative atomic structures of two Giardiavirus prototypes infecting G. duodenalis parasite
Source: PLoS Pathog. 2024 Apr 10;20(4):e1012140. doi: 10.1371/journal.ppat.1012140 (PMC11081498; doi:10.1371/journal.ppat.1012140)
Supplement: Attachment — Submitted filename: Point-to-point responses to review comments.docx [file ppat.1012140.s013.docx]

**Point-to-point responses to review comments**

**Reviewer #1:**

**Manuscript by Wang et. al describes the structures of two Giardia lamblia virus isolates belonging to the Totiviridae family. The two isolates GLV-HP and GLV-CAT have intracellular localization patterns. GLV-HP particles interact with each other and the possible reasons for the differences between the isolates is explored. The manuscript analyzes the differences between coat protein interactions at various interfaces and also compares the coat protein structures to other known Totiviridae virus structures. Overall, the structures are beautiful and the manuscript is well-written and easy to follow. I only have few major comments which will improve the overall impact of the manuscript.**

Thank you for your contributive comments. We truly concur that incorporating asymmetric reconstructions of RdRp(s) and the dsRNA genome in non-segmented T=1 dsRNA viruses would significantly improve the impact to the manuscript. However, this remains challenging, as recent studies by other research groups have not reported successful asymmetric reconstructions of the non-segmented T=1 dsRNA viruses (Ortega-Esteban et al., J Virol 2020; Stevens et al., mBio 2022; Grybchuk et al., Comm Biol 2022). Even so, we believe it is valuable to present the asymmetric reconstructions that we can achieve with our current datasets and the methods at our disposal.

Regarding the determination of asymmetric structures in large viruses, we have explored three primary methods, including those you have suggested. (1) Capsid subtraction method (e.g. Zhang et al., 2015 Nature) (2) Symmetry expansion/local reconstruction method (Colibus et al., Nat Comm 2019) (3) Symmetry relaxation method (e.g. Ilca et al., Nature 2019). We have already applied the capsid subtraction method and obtained typical spooled 2D-averaged classes of capsid-subtracted particles, but we are not able to build a 3D model (now shown in Supplementary Fig. S8). To address your comments, we have also applied the symmetry expansion/local reconstruction and the symmetry relaxation methods to our current datasets of GLV-HP and GLV-CAT, with the results are presented in a new Supplementary Fig. S7.

While the asymmetric reconstructions facilitated observation of some genome structures, determining the localization of CP-RdRp and the detailed genome organization remains difficult with the current datasets. The presence of one or a few RdRps in *Totiviridae* viruses, makes it hard to determine their structures. Additionally, *Totiviridae* viruses incorporate in a fused CP-RdRp form, possibly resulting in the non-fixed localization of RdRp within the virion due to weak or absent interaction between the CP and RdRp. In this manuscript, we have already presented and discussed new findings concerning the capsid and the capsid protein structures in *Totiviridae* and totivirus-like viruses, and we hope the revised manuscript is a satisfactory for the publication. We are enthusiastic about addressing the challenges of determining the RdRp/genome structures of T=1 dsRNA viruses in future research.

To address your additional comments, we have revised Fig. 3 and added Supplementary Fig. S2. The main text has been updated accordingly (Pages 13-14, Lines 261-282; Page 15, Lines 308-314).

**Major issues**

**1. How do the unmasked structures look and how is the genome organized inside the capsid. Providing a central slice of unmasked maps in the supplementary material is valuable.**

We have included a central slice of the unmasked maps for both GLV-HP and GLV-CAT in a newly added Supplementary Fig. S7 (Icos, Unmasked Models, Original).

**2. Did the authors observe any empty particles as observed in OmRV structures?**

While we did observe some empty particles in our study, they were fewer in number compared to those found in OmRV particles in our previous studies. Approximately 20% of the particles in both GLV-HP and GLV-CAT appeared empty, as described in method section (Page 23, Line 490). To provide a clearer picture, we have included additional raw micrographs for each in a new Supplementary Fig. S2.

**3. The authors only imposed icosahedral symmetry on the particles. Have the authors tried symmetry expansion and localized reconstruction of the five-folds to see if they can visualize any more details beyond whats observed from the icosahedral reconstruction.**

Beyond imposing icosahedral symmetry, we have also performed a symmetry expansion and local reconstruction around a 5-fold axis using cryoSPARC’s “Symmetry expansion” and “Local reconstruction” features. The results, displayed in a new Supplementary Fig. S7 (Sym Exp), reveal more asymmetric features compared to the icosahedrally-averaged reconstructions. However, these structures do not prominently display interior RdRp localization and still predominantly exhibit averaged layered genome structures.

**4. The newest version of cryosparc has a symmetry relaxation option and I can understand that the manuscript may have been submitted before the release. But it will be very useful to try this and see if we can see any features not visible in the icosahedral reconstruction. Especially for GLV-CAT because there are some features in the mask generated by cryosparc in FigS1.**

Following your kind suggestion, we utilized “Symmetry Relaxation” feature in the latest version of cryoSPARC to reprocess our datasets. The results, particularly for the GLV-CAT dataset, demonstrate some asymmetric genome features and are presented in a new Supplementary Fig. S7 (Sym Relax). However, as similar to the symmetry expansion method, these structures still do not display interior RdRp localization.

**5. Figure 3 is such an important and valuable figure but the color choices are not helpful to see the differences. This figure needs to be redesigned so that it can convey what the authors want the readers to understand. Please choose a color palette that is also friendly for color blindness.**

We have revised Fig. 3, considering your feedback and that of Reviewer #2. The figure now employs a color palette that is more accessible to those with color blindness, with a reduced color range and the use of blue and orange. We have also labeled the colored boxes as (a), (b), (c), and (d) to aid identification without relying solely on color differentiation. Furthermore, Fig. 3C has been added to provide clearer close-up views of surface loops.

**6. Please provide raw micrographs of both GLV-HP and GLV-CAT in the supplementary section.**

To offer a more detailed understanding of the proportion of empty particles and the aggregation of GLV-HP particles, we have included raw micrographs of both GLV-HP and GLV-CAT in a newly added Supplementary Fig. S2.

**Reviewer #2:**

**In this manuscript by Wang et al, provide high-resolution cryo-EM structures of two giardia lamblia virus (GLV) strains, GLV-HP and GLV-CAT. These dsRNA totiviruses infect, through an extracellular step, the parasite giardia lamblia. As there is interest in using these viruses as potential “virotherapy”, the high-resolution structures provided in this MS provide structural details that may prove critical in this regard. Furthermore, they expand our structural understanding of the Totiviridae family allowing for evolutionary analyses. While the structures are thus informative, the MS could be improved to provide more thorough analyses of the structures as in parts it is rather superficial.**

Thank you for your insightful suggestions to improve our manuscript. We have included new experimental nanoDSF thermographs (Supplementary Fig. S11) and a secondary-structure-based amino acid alignment (Supplementary Fig. S6) to deepen our discussion and address your comments. Fig. 2 and Fig. 3 have been revised following your guidance. The main text has been updated to reflect these new experiments and your feedback. Below are our responses to each of your major and minor comments.

**Major issues**

**1) The authors state that the GLV-CAT is “more stable” than GLV-HP (Ln 383) has this been experimentally determined or is this simply conjecture? If the latter, the authors should substantiate this claim by acquiring Tm of the different particles.**

Concerning the stability of GLV-CAT compared to GLV-HP, we agree that experimental verification is crucial. We have determined the inflection temperatures (Ti) of purified GLV-HP and GLV-CAT particles using Tycho nanoDSF, as shown in a new Supplementary Fig. S11. The melting points (Tm) correspond to these Ti values. The nanoDSF thermographs reveal several thermal dissociation/unfolding steps, with notable differences around 59.2^o^C and similarities around 75.0^o^C between GLV-HP and GLV-CAT. GLV-CAT displays an additional Ti value at 92.0^o^C. Although interpreting multiple dissociation steps in icosahedral particles can be challenging, as also reported in various icosahedral viruses (Yang et al., Engineering 2020; Rieser et al., J Pharm Sci 2020; Maus et al., Euro J Med Chem 2023). Nevertheless, these differences in thermal properties likely reflect distinct dissociation/unfolding steps for each GLV strain. In light of this, and to avoid overinterpretation, we have revised our description in the manuscript and refrained from using the term “more stable.” These revisions and the new Supplementary Fig. S11 can be found on Page 19, Lines 398-401.

**2) Is it possible to do mutagenesis (or perhaps it has already been done?) on the extra surface loops to validate their role in transmission? The authors should revise Fig 3 as it is rather difficult to interpret. More details should be provided regarding the amino acid identity within these loops. At present simply representing their secondary structure and amino acid numbers (lines 265-268) is too superficial.**

We acknowledge the importance of mutagenesis studies to validate the roles of the extra surface loops identified in GLV strains. However, a reverse genetic system for GLV is not yet available. The development of recombinant systems for non-segmented dsRNA viruses has still been limited, as evidenced by our recent work on creating an infectious DNA clone of the OmRV (Wang et al., Virology 2022). The mutagenesis of these loops involves a comprehensive examination of alterations or deletions in the amino acid residues across the five loops. From these points, the conducting mutagenesis assays are not feasible within a few months. We are motivated by our findings to develop a new recombinant system for GLV, aiming to complete mutagenesis studies within the next three years. We believe that our structural insights offer a foundation for future functional studies, potentially by other research groups as well.

In response to Reviewer #1 and your feedback, we have improved Fig. 3 for better clarity and interpretation.

Regarding the *Totiviridae* viruses (ScV-L-A, ScV-L-BC, GLV) and toti-like virus (OmRV), they have been aligned and classified based on the nucleotide sequences of the RdRp (Isawa et al., Virus Res 2011), but not based on the amino acid sequences of their CPs due to the diversity. To address this, we used PROMALS3D, a secondary-structure-based amino acid alignment tool, to generate an alignment of the phylogenetically distantly related CPs (Supplementary Fig. S6). This alignment displays well-conserved secondary structures across these viruses, indicating the loops 1, 2, and 3 in both GLV and OmRV CPs locate topologically similar regions (Supplementary Fig S6). It appears that the other extra surface loops are unique to the GLV or OmRV CPs. Furthermore, Fig. 3C now includes detailed views of these loops. We also examined the amino acid sequence homology and identity of these loops between GLV and OmRV CPs in the obtained alignment. While there may be some sequence homology in loop 3, no conserved motifs are apparent between the loops of GLV and OmRV (Supplementary Fig. S6). These findings and discussions have been added to Pages 13-14, Lines 261-282.

**Minor Issues**

**1) This is more of a philosophical question but the authors use “acquired” to describe the extracellular lifecycle of GLV (ln268, and elsewhere). Is it truly that they acquired it or did the other totiviruses/toti-like viruses “lose” this capability?**

The philosophical debate regarding whether GLV “acquired” or other Totiviridae viruses “lost” certain capacities is indeed complex and subjective. For clarity, we have opted to use the term “employed/employ” instead of “acquired” in certain parts of the manuscript (Page 5, Line 102; Page 14, Line 280). In other sections, we use “acquired” and “lost” together to present a balanced view of evolutionary changes (Page 13, Line 259; Page 25, Line 526).

**2) In Figure 2B, D, E, distances should be reported.**

We have updated Fig. 2 in the manuscript to include the distances.

**3) The Discussion needs to be expanded to contextualize the authors findings. It may be appropriate to move lines 221 -236 into the Discussion. Indeed, in many of the sections contain prose that would be better suited for the Discussion.**

Thank you for your suggestion. However, in our recent publications of showing new virus structures and discussing their acquired or lost structural features (e.g. Munke et al., mBio 2022; Wang et al., PLoS Pathog 2023), several reviewers suggested to integrate “Results” and “Discussion” sections. Consequently, we have adopted the “Results and Discussion” format in this manuscript, where each new structural finding is discussed in its respective section.

**4) The manuscript would benefit from a thorough editing for clarity as well as fixing grammatical and typos. In some cases, the word choice is inappropriate. Below are some examples but it is not an exhaustive list.**

**1. Ln 114: “methylguanocine” should be “methylguanosine”
2. Ln 147: a space is missing between duodenalis and Assemblage
3. Ln 149: The authors, I think mean “GLV-CAT does not form aggregates”.
4. Ln 159, ln 258 (and elsewhere): the use of “intensive” should be removed.
5. Ln 161 “lifestyle” is the wrong word choice.
6. Ln 178 “settled” is the wrong word choice.
7. Ln238 “relations” is the wrong word choice; perhaps “comparison”.
8. Ln293 “structurally collaborates” is wrong word choice**

We have thoroughly edited the manuscript for clarity and to rectify grammatical errors and typos including your list. Thank you for your valuable feedback, which has helped improve the quality of our manuscript.
