# Supplementary material for: High-resolution comparative atomic structures of two Giardiavirus prototypes infecting G. duodenalis parasite
Source: PLoS Pathog. 2024 Apr 10;20(4):e1012140. doi: 10.1371/journal.ppat.1012140 (PMC11081498; doi:10.1371/journal.ppat.1012140)
Supplement: Attachment — Submitted filename: Comments-to-reviewer-final-second revision.docx [file ppat.1012140.s014.docx]

**Point-to-Point Responses to Reviewer’s comments (Second revision)**

The revised manuscript by Wang et al is improved with respect to the original version. The comparative study of the two GLV isolates is interesting, and the results are informative to better understand the life cycle of these dsRNA viruses. The manuscript, however, lacks clarity at certain places, some of which are specified below. Also, it is important that the manuscript be seen by a native English speaker, as the narrative is sometimes ambiguous and difficult to follow. I raise some of the issues below, as well as comments on the Figures.

Thank you for your comments. We have addressed all your comments regarding main text and figures. Additionally, the manuscript has been proofread by another English editing service, recommended by our university, ensuring it has been reviewed by a native speaker.

**Main text**

Line 168-169: “the GLV-HP particles tend to interact with each other”: does this mean the purified particle preparation tends to aggregate? Or do they interact in a more specific pattern? please be more specific.

We made the sentence more specific in Line 168-171.

Lines 176-181 “Considering the predicted internal IRES sequence in the GLV genome, the translation of ORF1 (CP) does not initiate from the first methionine but from an internal amino acid residue [15,33]. The first amino acid residue of the CP was started from the internal Pro residue (PENIT …), according to a previous mass spectrometry analysis of the purified GLV particles (Supplementary Fig. S3)”
This sentence is not required, since the point is that the first visible residue in the structure is residue 70 or 71. It is interesting that translation of GLV CP does not begin with an AUG codon, but the sentence as written is confusing (“the first amino acid residue on the CP WAS STARTED from the internal Pro residue…”. It would be better to simply say that translation does not start with an AUG codon, but it is irrelevant for the results provided in this manuscript, and it could be rather explained in the legend to Figure S3 rather than in the main text, where it cuts the flow and leaves the reader wodering...

We omitted a large part of the text and simply wrote, “translation does not start with an AUG codon.” by referring to papers and Figure S3. Previous sentences were now in a figure legend of Supplementary Fig. S3.

Lines 187-197: this paragraph does not explain whether there is a CP core that superposes well between CP-A and CP-B (with an RMSD under 1Å, for instance), and whether there is a hinge between two or more domains that leads to different inter-domain orientations in A and B, but which could be superposed as individual rigid bodies (like the “carapace”, “apical” and “dimerization” domains defined for the in the internal CP of blue tongue virus (DOI: 10.1038/26694), or whether the large RMSD is distributed evenly throughout the molecule. Also, 4.3Å and 8.9Å RMSD differences in GLV-HP and GLV-CAT are quite substantial differences, and the reason for this does not stand out in Figure S4. This requires more explanation. Also, it is important to quote the number of C-alpha atoms used in the comparison that led to the quoted RMSD. The manuscript could be much more informative by taking these comments into account.

Thank you for clarifying this point. The A and B subunits are individual but chemically identical and orient in the same way. The subunits do not have obvious hinge regions. We have checked the superimposed CP-A and CP-B and calculated more carefully about the total RMSD and per-residue RMSD. The core domain apart from N and C-terminal, and red-circled regions in Fig. S4 are well aligned with 0.618 Å (684 Cα pairs of GLV-HP CP-A and -B), and 0.618 Å (732 Cα pairs of GLV-CAT CP-A and -B) while other loops and interfaces that contribute to the overall high RMSD value (4.338 Å across 775 Cα pairs in GLV-HP, and 8.863 Å across 848 Cα pairs in GLV-CAT). We have described the number of Cα atoms used in the comparison in the caption of Fig. S4 and Fig. S10.

Line 281: What is meant by “which have a non-exceptional intracellular transmission mechanism”, why is it “non-exceptional”?

The intended meaning was unclear in the previous sentence. We edited the sentence to “which only have an intracellular transmission mechanism” for clarity.

line 285: “Such as budding”. this term is normally used for enveloped viruses, which bud out from a cell by incorporating a patch of the cell membrane. But in the case of non-enveloped viruses such as GLV, it is important to explain what is meant by “budding”.

As you mentioned, GLV is a non-enveloped virus. It is still not fully revealed how they exit from the cell. We replaced “budding” with “particle release”.

Lines 398-400: “Nano differential scanning fluorimetry (nanoDSF) thermographs suggest differences in Ti (inflection temperature) values between GLV-HP and GLV-CAT particles”. Do these measurements “suggest” or show differences in Tm? If these are experimental measurements, they show that there are differences, not “suggest”.

These are experimental measurements. We replaced “suggest” to “show”.

Lines 414-417: “The structural differences between the two GLV prototypes show significant conformational differences in the C-terminal extension and the variable loop that are critical for CP–CP interactions”. Maybe it would better to say “`the comparative analysis of the two GLV prototypes reveal significant conformational differences”, rather than the redundant sentence “the structural differences reveal significant conformational differences”.

Thank you for the comment. The previous sentence was redundant as you mentioned. We edited the text as suggested.

**Figures:**
Figure 1, left panels. The transparent surface representation does not stand out. The squared frame does a better job.

Supposedly, you are commenting on Fig. 2. We replaced the surface representation with highlighting, which hopefully stand out.

Right panels: It would be useful to label some of the residues that are drawn in sticks and appear in all the panels, for instance, R923 (which should be colored according to atom type in panel E). They should be labelled at every instance in which they are displayed, not only in some panels (for instance, D927 is only labelled in panel B, whereas it appears to be present in D and E as well). This would provide the readers with way to better compare the various panels.

We thoroughly labeled the amino acid residues displayed in all panels and colored them by atom type.

In FIgure3, arrows pointing at the unique surface loops of GLV and OmRV would make it easier to see their location, rather than the transparent surfaces, which do not stand out. It would also be useful to have an overall RMSD of the superposition by for the common core of the two proteins. I presume the authors are comparing CP-A with CP-A (or CP-B with CPB) of each virus. This should be stated in the Figure legend.

The arrows are included in Fig. 3 now. The aligned structures shown in Fig. 3 were generated by pairwise structure-based alignment of eight CPs (CP-Bs) using TM-align and MUSTANG. All-to-all RMSD values present a total RMSD value of 5.4 Å between OmRV and GLV. Using the same method, we additionally calculated the total RMSD value of 5.8 Å only using only structure of GLV and OmRV CP-Bs (82% coverage of amino acid sequence). TM-score is 0.65 (> 0.5 assuming the same structural fold). A similar result (RMSD=5.6 Å, TM-score = 0.64) has been obtained if we use only the common core. The description has now been stated in the Lines 254-255. We used the CP-B of each virus for the comparison. The description has been added to the caption of Fig. 3.
